# Evidence for the Cost of Reproduction in Humans: High Lifetime Reproductive Effort Is Associated with Greater Oxidative Stress in Post-Menopausal Women

**DOI:** 10.1371/journal.pone.0145753

**Published:** 2016-01-13

**Authors:** Anna Ziomkiewicz, Amelia Sancilio, Andrzej Galbarczyk, Magdalena Klimek, Grazyna Jasienska, Richard G. Bribiescas

**Affiliations:** 1 Anthropology Unit in Wroclaw, Polish Academy of Sciences, Wroclaw, Poland; 2 Department of Anthropology, Yale University, New Haven, Connecticut, United States of America; 3 Department of Environmental Health, Faculty of Health Sciences, Jagiellonian University Medical College, Krakow, Poland; Universidade de São Paulo, BRAZIL

## Abstract

Life history theory predicts trade-offs between reproductive effort and maternal survivorship in energy-restricted environments. However, empirical evidence for the positive association between maternal mortality and reproductive effort from energetically challenged human populations are mixed and physiological mechanisms that may underlie this association are poorly understood. We hypothesized that increases in aerobic metabolism during repeated periods of pregnancy and lactation result in increased oxidative stress that may contribute to somatic deterioration, vulnerability to illness, and accelerated aging. We therefore predicted that lifetime gravidity and parity would be related to levels of biomarkers of oxidative stress, as well as antioxidative defence enzymes in post-menopausal women. Our hypothesis was supported by positive linear associations between levels of 8-OHdG, a biomarker of DNA oxidative damage (β = 0.21, p<0.05), levels of antioxidative defence enzyme Cu-Zn SOD (β = 0.25, p<0.05), and number of lifetime pregnancies. Furthermore, independent of age and health status, post-menopausal women with higher gravidity and parity (> = 4 pregnancies per lifetime) had 20% higher levels of 8-OHdG and 60% higher levels of Cu-Zn SOD compared to women with lower gravidity and parity (<4 pregnancies per lifetime). Our results present the first evidence for oxidative stress as a possible cost of reproductive effort in humans.

## Introduction

Humans, like all other organisms, face trade-offs between reproduction and maintenance when energy resources are limited [1]. Due to the metabolic costs of pregnancy and lactation, women bear significant physiological costs associated with reproductive effort and thus are susceptible to the negative consequences of these trade-offs [2]. Oxidative stress (OS), the state of imbalance between generation and management of damaging reactive oxidative species (ROS) and peroxidative agents through aerobic metabolism, is postulated as a central factor contributing to somatic deterioration with age [3]. Moreover, the intensity and effects of OS are influenced by environmental and life history factors. Among those factors, reproductive effort is hypothesized to be a key cost, contributing to increased OS throughout an organism’s lifespan [4]. Although this theoretical prediction has been extensively investigated in animals [5–6], compelling evidence is absent in humans.

Of the many sources of variation in oxidative stress, pregnancy, followed by lactation and childcare, are proposed to be central since they incur major increases in energy metabolism due to the costs of fetal development, milk production, and energy expenditure associated with maternal maintenance and physical activity [7]. This major rise in metabolism and energy flux might be associated with increased production of ROS and greater risk of oxidative stress [8].

The Disposable Soma Theory [9] predicts that in energetically constrained environments, organisms will benefit by investing limited resources in reproduction rather than in maintenance and repair. Over a lifetime, this leads to the accumulation of the cellular damage that impairs normal functioning of cells and tissues and contributes to faster somatic deterioration and aging [10]. Therefore, it can be expected that increases in energy metabolism related to high reproductive effort will contribute to maternal aging and lifespan reduction via accumulation of oxidative stress, especially in energetically constrained environments.

Up to date evidence in favor of these predictions in humans are mixed [11]. Studies of historical populations have been inconsistent, showing positive, negative, or no relationship between total parity and post-menopausal survival [12]. This inconsistency might be explained by several factors impossible to control for from archives data such as breastfeeding (constitutes the highest cost of single reproductive episode) or overall energy budget of woman [1,11]. Studies of contemporary populations have demonstrated a U-shaped relationship, with nulliparity and multipartiy (above 4 children) associated with highest mortality [12].

Significant increases in oxidative stress during gestation are evident, but only a few studies have investigated oxidative stress during the menstrual cycle [13–14], and virtually no studies have explored maternal oxidative stress during lactation [15]. Furthermore, the relationship between lifetime reproductive effort and accumulated oxidative stress in women has not been investigated.

To fill this gap, we conducted a study at the Mogielica Human Ecology Study Site [2], which includes 5 villages in the mountainous area of Southern Poland. During their reproductive period, women of this area have a broad range of parity (from 0 to 16 children) and intense physical activity associated with seasonally demanding agricultural work. Due to the mountainous terrain, operating heavy machinery is difficult and much of the work is done by hand. Previous studies by Jasienska and Ellison [16–17] documented very heavy physical activity levels during the summer season and light-moderate physical activity level during the winter season (according to the FAO/WHO/UNU1985 definition) [18] for women of reproductive age from this area. High reproductive effort accompanied by high energy flux during the reproductive period may intensify trade-offs incurred by reproductive processes later in post- reproductive life [1]. Indeed, previous studies conducted in this population demonstrated a reduction in maternal longevity in association with number of children [19].

Based on the recent observation of increased levels of biomarkers of oxidative stress in multiparous women suggesting that acute oxidative stress effects may accumulate over the consecutive reproductive episodes [20] we hypothesized that increased lifetime reproductive effort is associated with higher oxidative stress in postmenopausal women. To test this hypothesis in 100 postmenopausal, non-smoking women (median age 65 years) we measured urinary levels of three biomarkers: 8-Oxo-2'-deoxyguanosine (8-OHdG), copper-zinc superoxide dismutase (Cu-Zn SOD), and thiobarbituric acid reactive substances (TBARS) that together provide a composite assessment of whole body oxidative stress. These three biomarkers are widely and commonly deployed to assess oxidative stress in human studies [21–23]. In particular, levels of 8-OHdG depict the amount of oxidative damage accumulated in cellular DNA through the repaired lesions formed by ROS on the guanine base pair. Cu-Zn SOD represents the level of superoxide dismutase enzyme produced in cytoplasm, which contributes to the neutralization of ROS and serves as a main line of defense against ongoing oxidative stress. TBARS assessment reflects cellular lipid peroxidation as a result of oxidative stress.

## Methods

### Subjects

One hundred and twelve postmenopausal women were recruited from the general population of the villages during door-to-door visits to women’s houses. Postmenopausal women from all households in villages were invited to participate. Out of this number 12 women were excluded due to past and current smoking, which has been demonstrated to significantly impact oxidative stress biomarkers [24].

All women were examined by a physician on the day of sample collection to diagnose and exclude from participation any cases of ongoing bacterial or viral infection. Due to advanced age, over 70% of the postmenopausal women in the sample had long-term health problems resulting in past hospitalization or long-term use of medication. Vitamin supplementation was declared by about 30% of women. All women signed written consent for participation in the study. Study protocol was reviewed and approved by Bioethical Committee of the Jagiellonian University.

### General questionnaire and reproductive history

A general questionnaire was designed to collect information about participant’s age, level of education, marital status, current and previous employment and workload, long-term health problems, current and past use of medication, occurrence of diseases and medical conditions, lifestyle factors such as smoking and alcohol consumption, and reproductive history. Based on this information, the following reproductive effort variables were calculated: lifetime reproductive period (years between giving birth to the first and to the last child) and lifetime number of months spent pregnant and lactating. In addition, total energetic costs of reproduction were estimated based on data about pregnancy and lactation energetic requirements published by Butte and King [7]. Pregnancies that resulted in miscarriage were also included in these estimates, with the energy cost of the early-terminated pregnancy calculated for the first half of the normal pregnancy.

### Anthropometrics

Trained study assistants collected anthropometrical measurements of body height, sitting height, body mass, total and abdominal body fat, and body circumferences. These measurements were conducted during the initial visit to participants’ houses.

Body mass, body fat percentage, and abdominal fat rating were measured by bioelectrical impedance analysis (BIA) using a TANITA scale (model BC 545) with the accuracy to the nearest 0.1 kg (for body mass) and 0.1% (for body fat). Body height and sitting height were measured with a stadiometer, according to the standard methods, with the accuracy to the nearest 0.1 cm.

### Blood sample analysis

Trained nurses in the local outpatient clinic collected fasting blood sample from each participant. Samples were analyzed for basic blood parameters such as: hematocrit (HCT), hemoglobin (Hgb), red blood cells (RBC), platelet count, erythrocyte sedimentation rate (ESR), white blood cells (WBC), glucose concentration, (GLU) low and high density lipoprotein concentration (LDL and HDL) and CRP to assess general health status of participating women. All analyses were performed in the laboratory of local, nearby hospital in Limanowa. Evaluation of HCT, Hgb, RBC, MCV, WBC and platelet count was performed by an automated hematology analyzer (SYSMEX k-4500). ESR was determined automatically by Westergren method using SEDI 15. GLU, HDL, LDL and CRP concentrations were tested automatically using Pentra 400 analyzer by Horiba ABX.

### Urine samples analysis

Women collected a single first morning urine sample in sterile specimen containers provided by study assistants and delivered samples to the local outpatient clinic. All samples were collected during summer months (July and August) of 2013. Samples were aliquoted to separate vials and frozen within one hour of collection at -80C. Frozen samples were transported on dry ice to the Reproductive Ecology Laboratory in the Department of Anthropology at Yale University in order to conduct assay assessments of oxidative stress biomarkers. Samples were thawed and centrifuged for 10 minutes at 3000 G immediately before starting the assays to eliminate particulate matter that could interfere with the analyses. All assays were conducted using commercial ELISA kits in accordance to manufacturer directions and procedures. DNA damage EIA kit (Enzo Life Sciences, catalogue nr ADI-EKS-350) was used to measure levels of 8-OHdG. 8-hydroxy-2’- deoxyguanosine (8-OHdG), a modified nucleotide base and by-product of DNA damage that is excreted in the urine upon DNA repair. The sensitivity of the DNA Damage assay has been determined to be 0.59 ng/mL. The human Cu-Zn SOD enzyme-linked immunosorbent assay (Enzo Life Sciences, catalog nr ALX-850-033) was used to analyse level of Cu-Zn superoxidase dismutase. This enzyme, which is present in the cytoplasm, catalyses the dismutation of superoxide anion radicals to free oxygen and hydrogen peroxide. The assay sensitivity according to manufacturer protocol is 0.04 ng/ml. Levels of Thiobaturic Acid Reactive Substances (TBARS) were analyzed using TBARS Assay Kit (Cayman Chemical Company, catalogue nr 10009055) to determine cellular lipid peroxidation occurring as a consequence of oxidative stress.

All analyses were run in duplicates. Samples were randomly assigned to each assay. The average inter-assay variability for 8-OHdG, SOD, and TBARS analysis was 14% while intra-assay variability was 5%. All obtained results were corrected for urine concentration based on samples’ specific gravity.

### Statistical analysis

Values of oxidative stress and antioxidative defense biomarkers were log- transformed to insure a normal distribution. Gravidity was used as a composite measure of reproductive effort, which includes number of pregnancies, birth, and miscarriages and is highly correlated with time spent on lactation (see Results). Student’s t-test for independent sample was used to test the differences in health status, selected demographic and reproductive history variables between lower (<4 pregnancies) and higher (> = 4 pregnancies) gravidity group. Women’s assignment to these two groups was established based on results of several previous studies demonstrating increased costs of reproduction to mortality in women who gave birth to at least 4 children [12, 25–27].

General linear models (GLM) were used to test for the effect of gravidity on the level of oxidative stress and antioxidative defense biomarkers. Differences in biomarkers of oxidative stress between higher and lower gravidity groups were tested using Multivariate Analysis of Variance (MANOVA) and Covariance (MANCOVA). In these models log-transformed levels of 8-OHdG, Cu-Zn SOD and TBARS were entered together as the dependent variables because they all measure underlying concept of oxidative stress. Gravidity groups were used as the categorical independent variables. Age and abdominal fat were entered as the covariants, because both of these measures were previously shown to influence oxidative stress biomarkers [28]. Furthermore, selected blood parameters were entered to the models to account for participants’ health status. In particular, high glucose level and high ESR associated with increased and high HDL associated with decreased oxidative stress [28–30] were added to the models.

To test for linear association between gravidity and levels of oxidative stress biomarkers, linear regression models were used, with levels of the oxidative stress biomarkers entered as the dependent variables and gravidity entered as the independent predictors. Potential confounders (age, abdominal fat, GLU and LDL concentration, and ESR after 2 hours) were introduced as candidate covariates using backward step-wise regression methods with entrance and removal criteria of a p value of less than 0.05 and F value equal to 3.

In addition, as U-shaped association between postmenopausal mortality and parity was demonstrated in previous studies [11–12] we tested for the same shape of the association between oxidative stress biomarkers and gravidity using polynomial regression models.

All analyses were conducted using STATISTICA version 10 with p-values set on 0.05.

## Results

### Descriptive statistics and simple correlations

Average levels of oxidative stress biomarkers in the sample of postmenopausal women were 109.0 ng/ml (SD = 44.45) for 8-OHdG, 0.56 ng/ml (SD = 0.437) for Cu-Zn SOD, and 2.1 μM/g (SD = 1.75) for TBARS. As indicated by BMI (31.2; SD = 5.41) and percent of abdominal body fat (10.8; SD = 2.96), women participating in the study had good nutritional status. Although all of them were previously involved in occupational physical work, most of them did not work anymore, neither at their farms or houses nor outside of them. Thus the level of their current physical activity was relatively low. All descriptive statistics for women participating in the study are presented in Table 1.

**Table 1:** Blood parameters, anthropometric, demographic and reproductive characteristics of the study group.

	All women		Low gravidity		High gravidity	
	Mean	SD	Mean	SD	Mean	SD
**Age**	65.5	8.89	64.8	7.8	66.0	9.58
**Age at menopause (years)**	50.7	4.51	49.1	5.52	51.5	3.53
**Height (cm)**	155.7	6.36	156.3	5.85	155.3	6.70
**Weight (kg)**	75.9	14.91	73.5	15.51	77.6	14.39
**BMI (kg/m**^**2**^**)**	31.2	5.41	30.0	6.02	33.2	10.12
**Abdominal fat%**	10.8	2.96	10.2	3.23	11.2	2.72
**HCT (%)**	41.4	2.12	41.4	2.01	41.3	2.20
**Hgb (g/dL)**	14.1	0.86	14.1	0.86	14.1	0.87
**RBC (mln/μL)**	4.6	0.29	4.6	0.31	4.6	0.28
**ESR**	42.1	18.92	40.7	19.65	43.1	18.52
**WBC (tys/μL)**	2.0	0.55	2.0	0.51	1.9	0.58
**GLU (mmol/L)**	6.0	2.10	5.8	2.59	6.1	1.71
HDL (mmol/L)*	1.4	0.35	1.5	0.44	1.3	0.27
**LDL (mmol/L)**	3.6	1.04	3.7	0.95	3.5	01.10
**CRP**	3.9	13.80	2.3	2.51	2.67	2.71
**Education (years)**	8.9	3.03	9.5	3.13	8.5	2.90
**Past house work (years)**	39.7	16.76	37.4	18.05	43.6	13.96
**Past farm work (years)**	41.8	13.27	41.4	12.19	42.5	13.84
**Past other work (years)**	19.7	12.00	19.6	11.63	18.9	12.47
Lifetime pregnancy period (months)**	39.4	16.72	21.4	6.31	48.7	12.65
Lifetime lactation period (months)**	29.4	24.12	13.3	10.68	40.6	27.94
Lifetime reproductive period (years)**	10.4	5.60	6.1	3.69	12.9	5.01
Lifetime energy cost of reproduction (MJ)**	3688.8	2479.47	1753.8	855.00	3884.9	1897.09

Energetic costs of reproduction were estimated based on published values of energetic expenses of pregnancy and lactation [6]. Significant differences between gravidity groups asterisked

*p<0.05

**p<0.001.

Significant and positive correlations were found between levels of all oxidative stress biomarkers (r = 0.59, p<0.01 for ln 8-OHdG and ln Cu-Zn SOD; r = 0.31, p<0.01 for ln 8-OHdG and ln TBARS; and r = 0.33, p<0.01 for ln Cu-Zn SOD and ln TBARS).

No significant differences in oxidative stress biomarkers were found between women who did and did not report long-term health problems (hospitalization, long-term use of medication) (t = -1.91, p>0.05 for ln 8-OHdG; t = -0.73, p>0.1 for ln Cu-ZnSOD; t = 0.79, p>0.1 for ln TBARS). No significant differences were also found between women who did and did not supplement vitamins (t = -1.35, p>0.1 for ln 8-OHdG; t = -1.63, p>0.1 for ln Cu-Zn SOD; t = -0.31, p>0.7 for ln TBARS).

In contrast, significant associations were found between levels of oxidative stress biomarkers and health status parameters measured in blood. In particular, levels of TBARS were negatively associated with HDL (r = -0.25, p<0.05) and positively associated with GLU level (r = 0.27, p<0.01), while levels of 8-OHdG were negatively associated with ESR measured after 2 hours (r = -0.20, p<0.05).

Significant and robust positive correlations were found between gravidity and all investigated parameters of reproductive effort, namely between parity (r = 0.94, p<0.001), number of sons (r = 0.67, p<0.001), number of daughters (r = 0.65, p<0.001), lifetime lactation period (r = 0.57, p<0.001), lifetime reproductive period (r = 0.56, p<0.001) and number of miscarriages (r = 0.35, p<0.01). Furthermore, gravidity correlated negatively with age at first birth (r = -0.24, p<0.02) and positively with age at last birth (r = 0.40, p<0.001). Based on the above results, gravidity was chosen as a composite measure of reproductive effort.

### Oxidative stress in women with low and high reproductive effort

As expected, women with at least four pregnancies during a lifetime (higher gravidity) spent twice as long being pregnant and triple the amount of time lactating (t = -14.17, p<0.001 for number of months pregnant; t = -6.53, p<0.001 for number of months lactating). Consequently, they spent almost two times more energy on reproduction than women with lower gravidity, as indicated by estimated energetic cost of their pregnancies and lactations (t = -5.82, p<0.001). Differences in anthropometric, demographic, reproductive characteristics and health status parameters between women from lower vs higher gravidity group are presented in Table 1.

**Fig 1:**
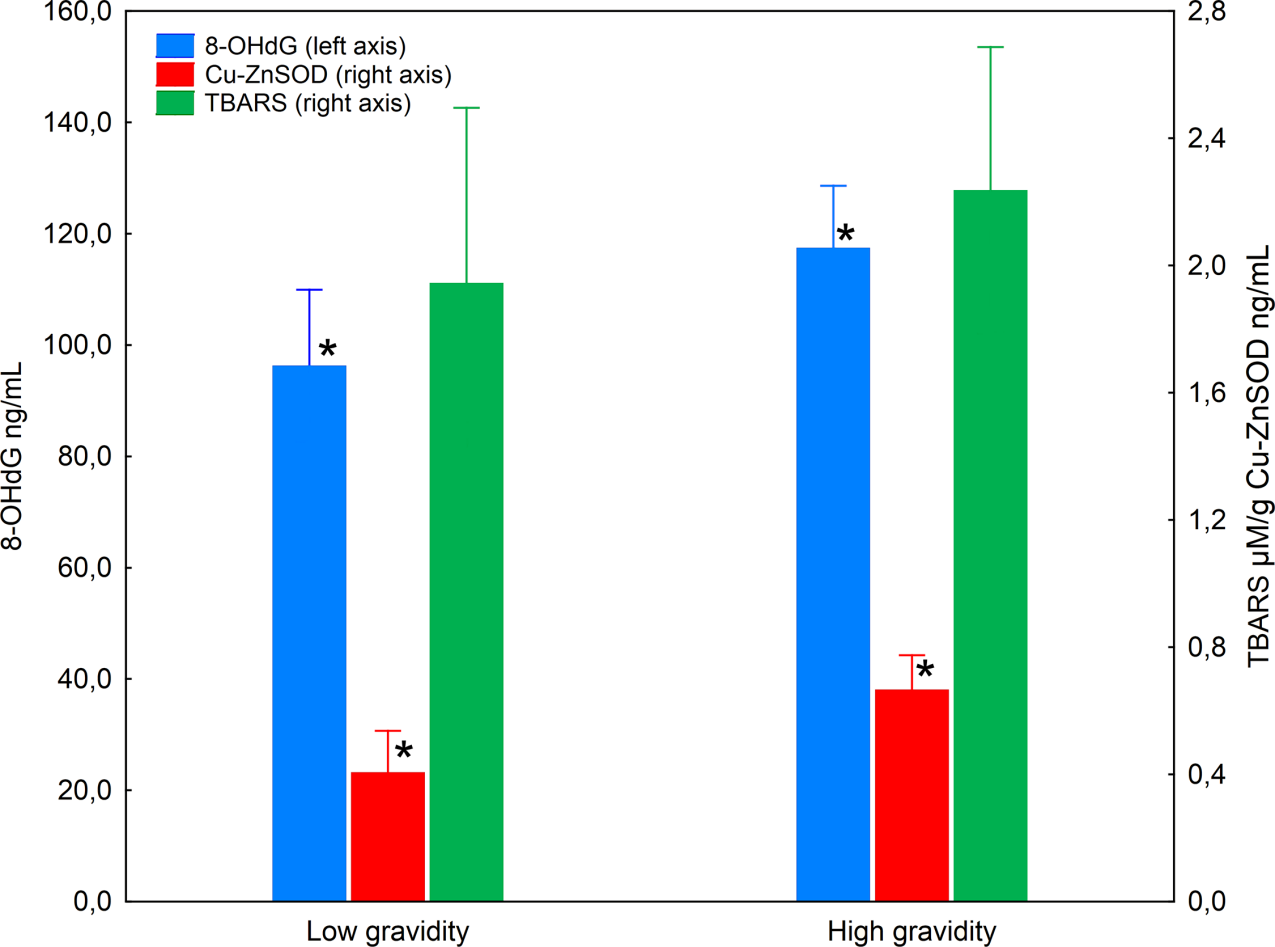
Levels of 8-OHdG, Cu-Zn SOD and TBARS in postmenopausal women from lower and higher gravidity group. Levels of 8-OHdG presented on left Y-axis, levels of Cu-Zn SOD and TBARS presented on right Y- axis. Significant differences between groups asterisked.

The multivariate results of MANOVA showed significant effect of low and high gravidity on the levels of oxidative stress biomarkers (Pillai’s Trace = 0.09, F = 3.28, p<0.05), indicating that consistent with our prediction, postmenopausal women with higher gravidity had higher levels of oxidative stress biomarkers than women with lower gravidity. In particular, the univariate F tests results showed significant difference between lower and higher gravidity group for log-transformed levels of 8-OHdG (F = 5.14, p = 0.025) and Cu-Zn SOD (F = 9.28, p = 0.003). No difference between those two groups was found for log-transformed TBARS levels (F = 2.24, p = 0.138) (Fig 1).

The observed differences in oxidative stress biomarkers remained significant when factors such as age and health status parameters (abdominal fat, ESR after 2 hours, GLU and HDL levels) were introduced to MANCOVA models (Pillai’s Trace = 0.09, F = 2.89, p<0.05). Consistently, higher gravidity was associated with significantly higher levels of log-transformed 8-OHdG (F = 5.38, p<0.05), and Cu-Zn SOD (F = 8.23, p<0.01). No significant difference between high and low gravidity group was found for TBARS levels (F = 0.82, p>0.3). Detailed results of MANCOVA model are presented in Table 2.

**Table 2:** Univariate results of MANCOVA models of the association between biomarkers of oxidative stress and reproductive effort adjusted for age and health status parameters.

	8-OHdG (ln)		Cu-Zn SOD (ln)		TBARS (ln)	
	F	p	F	p	F	p
**Age**	0.005	0.943	0.25	0.613	0.02	0.899
**GLU**	1.67	0.199	1.97	0.164	8.82	0.004
**ESR**	1.86	0.176	0.38	0.541	0.18	0.670
**HDL**	0.03	0.861	0.19	0.659	6.29	0.014
**Abdominal fat**	4.13	0.045	0.01	0.929	0.68	0.410
**Gravidity group**	5.38	0.023	8.23	0.005	0.82	0.368

### Association between biomarkers of oxidative stress and reproductive effort

**Fig 2:**
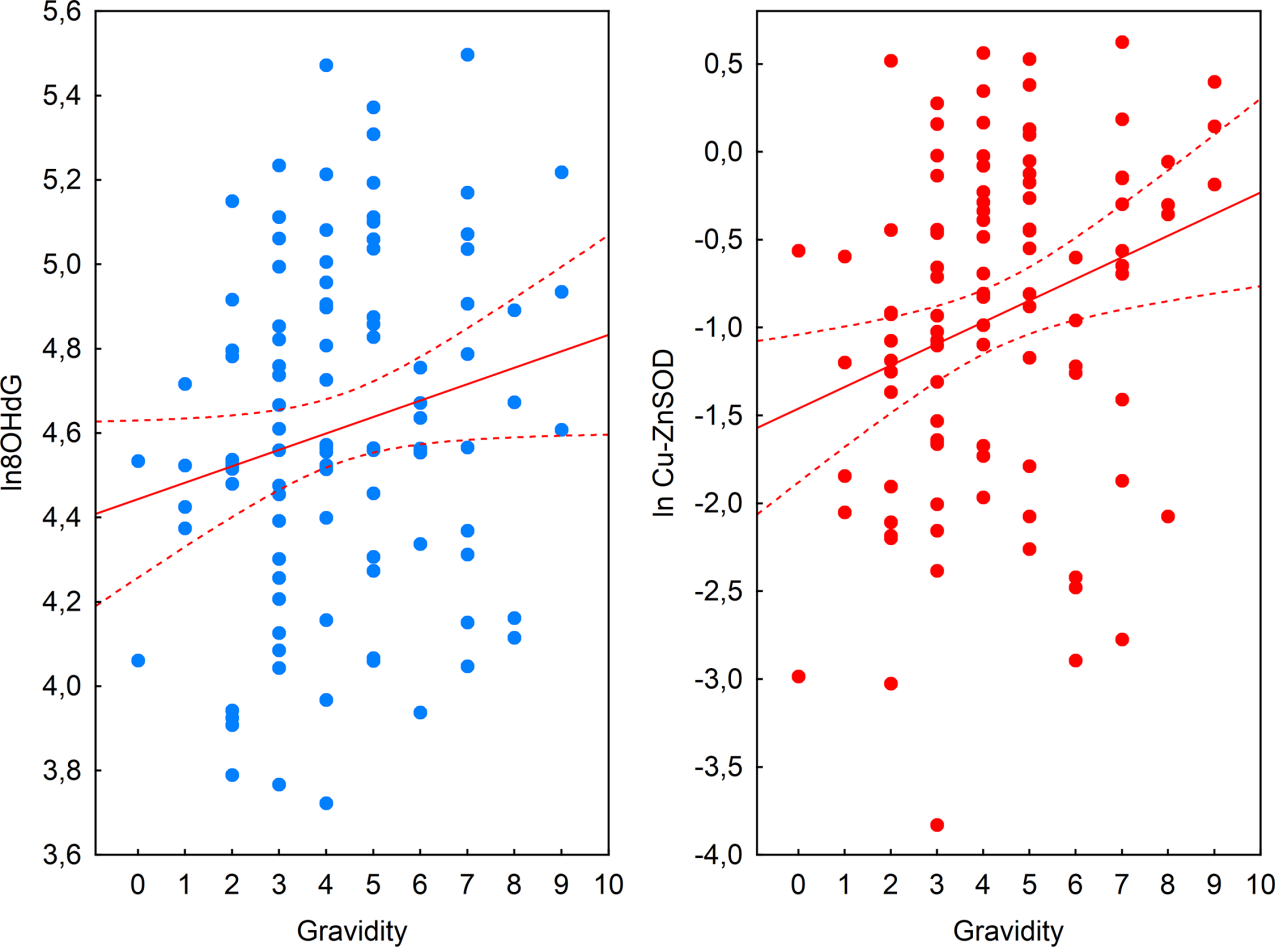
Positive associations between gravidity and levels of oxidative damage and defence biomarkers. Association between gravidity and 8-OHdG (a) and Cu-Zn SOD (b).

Simple regression models confirmed predicted positive association between biomarkers of oxidative stress and gravidity for log-transformed values of 8-OHdG (β = 0.19, p = 0.05), Cu-Zn SOD (β = 0.27, p<0.01) and TBARS (β = 0.21, p<0.05). Furthermore, results of backward stepwise multiple linear regression models with age and health status parameters (abdominal fat, GLU, ESR, HDL) evidenced independent effect of gravidity on the levels of 8-OHdG (R^2^_adj_ = 0.06, p<0.02) and Cu-Zn SOD (R^2^_adj_ = 0.05, p<0.02). Final model for ln 8-OHdG included abdominal fat (β = -0.26, p<0.02) and number of pregnancies (β = 0.21, p<0.05) and for ln Cu-Zn SOD only number of pregnancies (β = 0.25, p<0.02) (Fig 2). Although results of stepwise regression analysis for TBARS were statistically significant (R^2^_adj_ = 0.14, p<0.01) gravidity was removed from the model in the third step (F_remov_ = 0.97, p>0.3) leaving GLU and HDL as the best predictors of ln TBARS in the model (β = 0.28, p<0.01 for GLU and β = -0.25, p<0.02 for ESR).

Results of second order polynomial regression analysis showed no evidence for U-shaped association between gravidity and levels of oxidative stress biomarkers. Detailed results of the analysis are presented in Table 3.

**Table 3:** Results of second order polynomial regression for biomarkers of oxidative stress and gravidity.

	R^2^_adj_	p	t	p	β
**ln 8-OHdg**	0.03	0.095			
**Intercept**			25.91	<0.001	
**Gravidity**			1.45	0.151	0.56
**Gravidity**^**2**^			-0.97	0.333	-0.37
**ln Cu-Zn SOD**	0.06	0.020			
**Intercept**			-4.50	<0.001	
**Gravidity**			1.43	0.156	0.54
**Gravidity**^**2**^			-0.75	0.457	-0.28
**ln TBARS**	0.05	0.035			
**Intercept**			-0.77	0.441	
**Gravidity**			2.04	0.044	0.78
**Gravidity**^**2**^			-1.54	0.127	-0.59

## Discussion

To our knowledge, this is the first study that clearly demonstrates higher oxidative stress in association with elevated lifetime reproductive effort in women. We found that women with higher lifetime gravidity had nearly 20% higher levels of 8-OHdG, a biomarker of oxidative damage to DNA, and 60% higher levels of Cu-Zn SOD, a biomarker of antioxidative defense compared to women with lower gravidity. Moreover, levels of 8-OHdG and Cu-Zn SOD exhibited a linear association with lifetime gravidity suggesting a dose dependent response between reproductive effort and oxidative stress.

Increased level of DNA oxidation in women with higher gravidity reflects an imbalance between ROS production and ROS-mediated damage repair, and indicates elevated oxidative stress in response to increased reproductive effort. Similarly, higher levels of Cu-Zn SOD, the front-line defense enzyme against reactive oxygen species-mediated injury, indicates higher ROS production and should be also interpreted as a biomarker of increased oxidative stress [22]. While these results might also be construed as reflecting acute differences in oxidative stress between high and low gravidity women, the lack of differences in lifestyle variables associated with oxidative stress makes this unlikely. Instead, we offer that the cumulative effects of reproductive effort contribute to greater susceptibility to oxidative stress in post-menopausal women.

In accordance with our results, other studies have shown that levels of oxidative stress biomarkers increase, while antioxidant capacity decreases, during pregnancy and when compared to non-pregnant status [31–33]. Moreover, oxidative damage associated with pregnancy may accumulate with consecutive pregnancies. Oxidative stress index is more than 50% higher and total antioxidant capacity is about 10% lower in cord blood of multiparous women when compared to primiparous women [20].

Elevated oxidative stress observed in women with higher gravidity might be attributed to higher energy metabolism associated with the reproductive process. Indeed, as shown in our sample women with higher gravidity spent over two times more energy on reproduction when compared to women with lower gravidity. On average, daily energy requirements rise by about 20% during woman’s pregnancy and up to 50% during lactation [7]. During pregnancy, this additional energy is utilized to facilitate foetus tissue development and metabolism. In late pregnancy, about 55% of foetal total glucose utilization is used for oxidation [34]. In addition, maternal carbohydrate oxidation increases about 30% when compared to postpartum period [35]. Thus, amplified oxidation during gestation may create an extra burden to maternal oxidative/antioxidative balance and result in increased oxidative stress.

Although convincing evidence does not exist for women, studies in post-partum cows imply gradual increases in oxidative damage and decreases in antioxidant capacity during the early process of lactation. These effects on females’ oxidative/antioxidative profiles tend to intensify during the lactation of consecutive offspring [36–37]. Lactation periods in red squirrels were associated with an almost 50% increase in daily energy expenditure (DEE), resulting in a 2-fold increase in oxidative damage compared to the non- lactating period [38].

During lactation in humans, surplus energy is used for breast milk synthesis but glucose oxidation does not differ between lactating and non-pregnant, non-lactating women [39], which suggests that other sources of fuels are utilized during this period. Indeed, several studies report high lipid mobilization during lactation [40]. In contrast to glucose, which is a primary source of free radical production, lipids metabolism produces significantly less ROS [41]. This difference in fuels utilization may explain why the number of pregnancies but not periods of lactation was associated with oxidative damage and antioxidative defense in postmenopausal women in our study.

High reproductive effort might lead to oxidative stress and accumulation of the oxidative damage, especially when accompanied by elevated physical activity levels that may limit the energy available for repair mechanisms during the reproductive period [9]. Women from our rural population devoted a considerable amount of energy to intensive physical work during their reproductive period as shown by previous studies [16–17]. Seasonal increases in workload led to a reduction in levels of ovarian steroids, which suggests that energy expenditure associated with physical activity constituted a biologically significant constraint on women’s energy budget. On the average, mean energy expenditure for women of reproductive age in this population was 10.7 MJ/day. During August, a month with the most demanding physical work, mean total daily energy expenditure was 2.02 (expressed as a multiple of BMR) [16]. Although acute physical activity may constitute independent and additional source of oxidative damage, [42] it is unlikely that physical activity at present had any significant effect on oxidative stress, as most of the participating women were unable to maintain high physical activity due to their advanced age. Instead, we propose that high physical activity during the reproductive period in addition to high reproductive energy investment might disturb oxidative/antioxidative balance and generate oxidative damage.

Recently published, critical review on oxidative stress as a cost of reproduction by Speakman and Garratt [6] concluded that in order to magnify the allocation trade-off more studies should be conducted in females that are highly challenged during their reproduction by energetically costly environmental burdens and in females that engage in repeated reproductive attempts. Our sample of postmenopausal rural women from Poland fulfills both criteria. We demonstrated the positive association between oxidative stress and reproductive effort, that may evidence the trade-off, in individuals under high environmental pressure associated with seasonal changes in physical workload and diet, engaging in several (up to 10) consecutive reproductive episodes. It is however possible, that the observed variation in oxidative stress may result from completely different source than the trade-off itself. For instance, antagonistic pleiotropic effect of genes may play a role here as evidenced by our recent study on *ApoE4* allele [43]. The *ApoE4* allele (at the *ApoE* locus, encoding apolipoprotein E) is associated with significantly increased risk of poor health. We demonstrated that women who carry at least one *ApoE4* allele had significantly higher level of progesterone than women without *ApoE4* allele. This result indicates that higher fertility in women might be associated with increased risk of poor health and possibly higher oxidative stress via genetic pathway. Moreover, psychosocial stress associated with trauma episodes during life may also influence level of oxidative stress [44]. Thus future studies investigating the association between oxidative stress and reproductive effort should take these factors into account.

The biological significance of the oxidative stress difference between high and low gravidity remains to be determined. However higher levels of oxidative stress biomarkers are associated with greater mortality in older individuals after controlling for smoking, alcohol consumption, and other factors that are commonly linked to decreased lifespan [45]. Moreover, Semba et al. [46] demonstrated that oxidative stress is associated with increased mortality at older ages in women. Although these investigations used different biomarkers e.g. reactive oxygen metabolites (d-ROM), total thiol levels (TTL) and protein carbonyls, their results suggest that the differences in oxidative stress biomarkers found in our study might be indicative of greater risk of decreased lifespan due to accelerated aging.

Life history theory predicts trade-offs between reproductive effort and longevity when energy resources are limited [47–48]. Accordingly, lifetime parity was demonstrated to shorten lifespan in some but not all energetically constrained environments [19, 49–52]. Lifetime parity and gravidity has been also observed to be a risk factor to many diseases associated with higher mortality in post reproductive life such as cardiovascular disease, some forms of cancer, insulin resistance, diabetes, and Alzheimer’s disease [53–56]. Oxidative stress is implicated to play central role in pathophysiology of these diseases [57–58]. Our study links these phenomena by presenting evidence for the oxidative stress as a possible physiological cost of high reproductive effort in humans.
